# ID 68 - Projeto ACERTO: Impacto no Tempo de Internação, Cuidados Perioperatórios e Custos Hospitalares

**DOI:** 10.5327/2237-9622.2025.v34s1.68

**Published:** 2025-11-25

**Authors:** Deyvid Fernando Mattei da Silva, Deyvid Fernando Mattei da Silva, Laís Maria de Campos, Otávio Monteiro Becker

**Keywords:** protocolos clínicos, alta do paciente, custos hospitalares, assistência perioperatória, urologia

## Abstract

**Introdução:**

O projeto ACERTO (Aceleração da Recuperação Total no Pós-Operatório) foi criado em 2004 como um projeto de pesquisa no Brasil, inspirado pelos protocolos fast-track e pelo programa europeu ERAS (). Implementado em 2005, o ACERTO introduziu uma abordagem multimodal voltada para otimizar os cuidados perioperatórios, com foco na aceleração da recuperação dos pacientes submetidos a diversos tipos de cirurgias. O protocolo trouxe adaptações para a realidade latino-americana e ampliou sua aplicação para diferentes especialidades cirúrgicas, promovendo benefícios até em cirurgias de menor porte. Este estudo tem como objetivo comparar o tempo de internação, cuidados perioperatórios e custos hospitalares de pacientes submetidos a cirurgias urológicas antes e após a implementação do projeto ACERTO, no período de 2018 a 2022, em hospital público em São Paulo, referência em saúde do homem.

**Método:**

Para a implementação do projeto foi utilizado o método Breakthrough, que é composto por quatro fase: Coleta de dados inicial, elaboração do plano de mudança, coleta de novos dados após a implementação e análise dos novos dados e comparação com os dados iniciais. Para calcular os custos hospitalares, foi utilizada a metodologia de macrocusteio, neste estudo foram analisadas as diárias de internação.

**Resultados:**

A implementação do projeto ACERTO resultou em melhorias significativas nos desfechos clínicos e financeiros dos pacientes submetidos a cirurgias urológicas. Houve redução substancial no tempo de internação em diversas cirurgias. Na ressecção transuretral de próstata, o tempo médio de internação diminuiu de três para um dia, enquanto a ressecção transuretral de bexiga de dois para um dia. Procedimentos como a prostatectomia diminuíram de cinco para dois dias, e a nefrolitotripsia percutânea de quatro para dois dias. A ureterolitotripsia também mostrou redução, passando de dois para um dia de internação. Os custos hospitalares também foram impactados positivamente. O número total de dias de internação foi reduzido de 9.365 em 2018 para 4.542 em 2022, enquanto os custos totais tiveram um decréscimo de R$ 34.968.136,92, valores corrigidos para 2022, para R$ 10.446.600,00 no mesmo período, correspondendo a uma economia de 70%. Além disso, houve melhorias nos cuidados perioperatórios, a dose correta de antibiótico profilático foi administrada corretamente em 98% dos casos em 2022, comparado a apenas 50% em 2018. A incidência de dor nas primeiras 24 horas após a cirurgia reduziu de 25% para 5%, e o tempo médio de jejum total passou de 22 para 16 horas.

**Conclusão:**

Com base na análise dos resultados obtidos pela implementação do Projeto ACERTO, observou-se melhoria substancial tanto nos desfechos clínicos quanto nos financeiros, corroborando os resultados de outros estudos. O ACERTO demonstrou ser altamente eficaz na redução dos custos hospitalares e do tempo de internação, bem como na melhoria da recuperação pós-operatória em diversos contextos cirúrgicos. A redução dos custos hospitalares, do tempo de internação e das complicações pós-operatórias reflete o sucesso da abordagem multimodal proposta pelo protocolo. Seja em cirurgias eletivas, de emergência ou de grande porte, o ACERTO demonstrou ser uma estratégia eficaz para otimizar os cuidados perioperatórios, acelerar a recuperação dos pacientes e aliviar a pressão sobre o sistema de saúde pública.

